# Impact of Brushing Protocols on Dentin Abrasion Caused by Different Abrasive Slurries

**DOI:** 10.3290/j.ohpd.b1694095

**Published:** 2021-07-15

**Authors:** Giulia L.S. Krol, Peter Wetselaar, Thomas Attin, Chryssa E. Papagianni, Florian J. Wegehaupt

**Affiliations:** a Staff Member, Clinic of Conservative and Preventive Dentistry, Center of Dental Medicine, University of Zürich, Zürich, Switzerland; Department of Oral Kinesiology, Clinic of Orofacial Pain and Dysfunction, Academic Center for Dentistry Amsterdam (ACTA). Performed the experiments, wrote the manuscript.; b Clinic Head and Associate Professor Department of Oral Kinesiology, Clinic of Orofacial Pain and Dysfunction, Academic Center for Dentistry Amsterdam, Amsterdam, the Netherlands. Proofread the manuscript.; c Professor and Director, Clinic of Conservative and Preventive Dentistry, Center of Dental Medicine, University of Zürich, Zürich, Switzerland. Research idea, experimental design, proofread the manuscript.; d Staff Member, Department of Oral Kinesiology, Clinic of Orofacial Pain and Dysfunction, Academic Center for Dentistry Amsterdam, Amsterdam, Netherlands. Proofread the manuscript.; e Head of Division of Preventive Dentistry and Oral Epide Miology, Clinic of Conservative and Preventive Dentistry, Center of Dental Medicine, University of Zürich, Zürich, Switzerland. Research idea, hypothesis, experimental design, contributed substantially to discussion and writing the paper, proofread the manuscript.

**Keywords:** brushing, continuously, dentin wear, intermittently

## Abstract

**Purpose:**

To determine the impact of the brushing protocol on dentin wear by comparing continuous to intermittent brushing, with the same total time of brushing.

**Materials and Methods:**

Dentin specimens (n = 120) were evently distributed into six groups (A–F). The samples were brushed with slurries of different relative dentin abrasivity (RDA): Groups A+B (Sident 2480-1; RDA 85), groups C+D (Zeodent 113; RDA 67), and groups E+F (Zeodent 103; RDA 174). Groups A+C+E were brushed continuously (25 min) with one slurry preparation, while groups B+D+F were brushed intermittently (25 x 1 min) with a renewal of the slurry after each sequence. Dentin wear was determined using surface profilometry and statistically analysed with ANOVA and post-hoc tests.

**Results:**

Neither the mode of brushing (p = 0.72) nor the interaction (p = 0.18) of the brushing mode with the type of abrasive particles had a significant influence on the abrasive dentin wear. Only the type of abrasive particles had a statistically significant influence on abrasive dentin wear (p < 0.001).

**Conclusion:**

The mode of brushing (continuously or intermittently) has no influence on abrasive dentin wear.

Dental hard tissue loss caused by toothpastes has gained increased interest in modern dentistry. Today, a multitude of dental healthcare products are on the market which aim to avoid or reduce abrasion and/or erosion,^8^ In addition to individual habits of brushing, abrasion caused by toothpastes has been shown to be significantly related to hard tissue loss.^10^

In 1976, the gold standard for determining dental hard tissue loss caused by toothpastes, the so-called relative dentin abrasivity method (RDA), was developed. RDA is a well-established method; its principals were adopted by both the American Dental Association and the International Standard Organisation.^7^ It is considered a useful tool for determining the relative abrasivity level of toothpastes and abrasive powders.^5^ While performing RDA measurement, radioactive irradiated root dentin is brushed with a slurry prepared from the tested toothpaste and water/artificial saliva or the reference abrasive material under standardized conditions. During this procedure, radioactive dentin particles are released into the slurry. The radioactivity of the test slurries is then compared to the radioactivity of the reference abrasive slurry, which is given a value of 100. Based on this, the RDA the test slurries is quantified. ISO 11609^7^ defines that, with each slurry, it takes a minimum of 3000-6000 brush strokes (corresponding to about 25 min) before a reliable statement on its abrasiveness can be made. One might assume that during 25 min of continuous brushing, the shape and size of the abrasive particles in the slurry as well as the pH of the slurry might change. But in daily life, patients do not brush their teeth continuously for 25 min. In fact, they brush for much shorter periods (2 min)^4^ and always use fresh toothpaste each time. Therefore, one might assume that the abrasivity measured by brushing continuously may not reflect the abrasivity of a toothpaste used by patients under daily conditions.

Therefore, the aim of the present study was to determine if brushing continuously with the same slurry causes less abrasion than brushing intermittently, replacing the slurry every minute. The null hypothesis was that brushing intermittently does not cause more dentin wear than does continuous brushing.

Additionally, throughout this study, the shape of the abrasive particles was determined using scanning electron microscopy (SEM), and the pH was monitored to determine whether it changed.

## Materials and Methods

### Specimen Preparation

In total, 120 specimens were prepared from the roots of 20 permanent non-damaged bovine incisors. Teeth were stored in tap water (Zürich, Switzerland, no added fluoride) until use. From each root, six cylindrical dentin specimens (3 mm diameter) were obtained by drilling dentin cores out of the root with a diamond trephine mill (BFW 40/E, Proxxon; Föhren, Germany) under water cooling applied perpendicular to the long axis of the root. All specimens from a given root were marked with the letters a–f and the respective root number to facilitate sample allocation. To ensure correct positioning in the brushing machine and to further facilitate profilometry analysis, the dentin specimens were centrally embedded in acrylic resin, using cylindrical silicone molds (Paladur, Heraeus Kulzer; Hanau, Germany).

After embedding the specimens, the acrylic/dentin cylinders were ground flat in two steps, using an automated grinding machine (Struers, Tegramin-30; Ballerup, Denmark) under constant water cooling and a pressure of 5 N. To grind down the acrylic/dentin cylinder surfaces, carborundum disks (Waterproof Silicon Carbide Paper, Struers) with decreasing grain size (4000 grit, 2000 grit) were used, resulting in a flat dentin area. To align the polished dentin surfaces parallel to the bottom of the embedded samples, the back of the samples was milled down using a mill drill (BFW 40/E, Proxxon; Föhren, Germany) resulting in a uniform sample thickness of 3 mm.

Finally, the six specimens (a–f) of each bovine root were evenly distributed into the respective six experimental groups (A–F, n = 20). To avoid dehydration at all times, the specimens were stored in 100 ml tap water until further use, but for no longer than a week, before the water was renewed (Zürich, Switzerland, no added fluoride).

The experimental procedure is presented in Table 1.

**Table 1:** Sample allocation and experimental procedure

1. Preparation of six dentin samples (a–f) each from 20 bovine lower incisors					
2. Allocation of samples to six groups					
Group A all ‘a’ samples	Group B all ‘b’ samples	Group C all ‘c’ samples	Group D all ‘d’ samples	Group E all ‘e’ samples	Group F all ‘f’ samples
(n = 20)	(n = 20)	(n = 20)	(n = 20)	(n = 20)	(n = 20)
3. Preconditioning of samples (1000 brush strokes)					
4. Recording baseline surface profiles					
5. Continuous toothbrush abrasion (2.5 N; 120 brush strokes/min; 25 min; PARO M43)					
Sident 2480-1	–	Zeodent 113	–	Zeodent 103	–
6. Intermittent toothbrush abrasion (2.5 N; 120 brush strokes/min; 25 x 1 min; PARO M43)					
–	Sident 2480-1	–	Zeodent 113	–	Zeodent 103
7. Recording final surface profiles to determine dentin wear					

### Preparation of Slurries

In total, three experimental slurries were prepared. The abrasive particles (all of which consisted of precipitated SiO_2_) used for the slurries were Sident 2480-1 (Sident, Evonik Degussa; Essen, Germany) for groups A and B, Zeodent 113 (Zeodent 113, Evonik Degussa) for groups C and D and Zeodent 103 (Zeodent 103, Evonik Degussa) for groups E and F. To prepare the slurries, 90 g of the respective abrasive were mixed with 450 g of a hydroxyethyl-cellulose glycerine mix and 0.45 g of silicon antifoam (Silicone Antifoam, Sigma-Aldrich; St Louis, MO, USA). The hydroxyethyl-cellulose glycerine mix was prepared by mixing 200 g of glycerine (glycerine, Thommen Furler; Rüti, Switzerland), 792 g of artificial saliva (for formulation, see Bächli et al^3^) and 792 g of ultrapure water (TKA MicroPure, Huber Lab; Niederelbert, Germany) and heated up to 70°C. When a temperature of 70°C was reached, 10 g of hydroxylethyl-cellulose (Sigma-Aldrich) was added to the solution under constant mixing. The solution was then set aside to cool down to room temperature. Next, 206 g of 1.62% Na-hydrogencarbonate solution (Merck; Darmstadt, Germany) was added to the rest of the solution and and mixed.

The RDA of the slurry used in groups A and B (Sident 2480-1) was 85, in groups C and D (Zeodent 103) it was 67, and in groups E and F (Zeodent 113) RDA was 174.

### Brushing Procedure

Before brushing the specimens with the test slurries, a pre-conditioning phase was performed, in which the specimens were brushed for 1000 strokes, 120 BS (brush strokes)/min and a standard slurry. To produce the standard slurry, 20 g of toothpaste (Elmex, Gaba Schweiz; Bern, Switzerland) and 40 g of artificial saliva were put in a cup and mixed thoroughly for 5 min.

Before starting the experimental brushing procedures, two parallel reference lines were scratched in the embedding resin outside the dentin specimen and then covered with adhesive tape to prevent them from being damaged during brushing. Brushing was performed in an automatic brushing machine with reciprocating movements at a frequency of 120 BS/min.

For each specimen, a medium bristle stiffness toothbrush (ParoM43, Esro; Zürich, Switzerland) was used. A constant brushing force of 2.5 N was applied by fixing a weight on the toothbrush and regularly checked with a spring scale.

The specimens of groups A, C and E were brushed continuously (1 x 25 min) with the respective slurries, while the specimens of groups B, D, and F were brushed intermittently (25 x 1 min). In groups A, C and E, 3 ml of the respective slurries were applied once. In groups B, D and F the slurry was renewed every minute.

As it was the aim of the study to evaluate continuous vs intermitting brushing, brushing and testing conditions did not have to be consistent with the ISO standards for testing toothpastes.

### Profilometry Assessments

Baseline profiles were recorded after preconditioning of the specimens, but before starting the test brushing procedures. They were used as a reference to later calculate the dentin wear.

For each specimen, five baseline profiles spaced at 250 µm over an area of 1 mm width along the entire length of the specimen were recorded using a stylus profilometer (Perthometer S2 Concept, Mahr; Göttingen, Germany), with a stylus force <0.7 mN and a lower measuring limit <130 nm profile difference.^15^ To achieve stable profilometric readings, the specimens were measured under wet conditions.^2^

After finishing the respective brushing procedures, new surface profiles (final profiles) were recorded. To ensure an exact repositioning of the specimens during the pre- and postbrushing recording of the surface profiles, the profilometer is equipped with a special custom-made jig. Custom-made software superimposed the initial (baseline) and the final profiles. Superimposition was possible by overlaying the reference points (parallel lines in the acryl resin). The difference in step-height between baseline and final profiles yielded the abrasive dentin wear value. The measurement limits of the profilometer was 0.1 µm.^2^

In cases of assessed dentin wear below this limit, the respective profile value was set to 0.000 µm.^2^

The average value of the five respective profiles was considered as loss of dentin due to the brushing procedure per sample.

### pH Measurement

To determine changes in pH of the slurries during brushing, the pH of all the slurries was measured at baseline, after 1 min and after 25 min of brushing. For this experiment, freshly prepared slurries were used. The pH was measured using a 780/781 pH-/ion meter device (Metrohm; Zofingen, Switzerland).

### Scanning Electron Microscopy

To determine the shapes of the abrasive particles and whether they changed during brushing procedures, SEM image were examined. This was performed with slurry collected at baseline, after 1 min and after 25 min of brushing.

To separate the abrasive particles from the slurries, the slurries were placed in a centrifuge tube and mixed with ultrapure water (TKA MicroPure, Huberlab; Niederelbert, Germany) until a weight of 45 g was reached.

After adding the ultrapure water into the tubes with the respective slurries, the tubes were centrifuged (Heraeus Megafuge 8R, Thermo Fisher Scientific; Waltham, MA, USA) for 3 min at 2000 rpm. Centrifuging was repeated four times. After centrifuging each time, the supernatant water was suctioned off and centrifuging was again performed until the water was visibly completely clean.

Finally, two more rounds of centrifuging runs were performed, adding 20 ml of ethanol into the centrifuge tube. To dry the abrasive particles, the centrifuge tube was placed in a digital incubator (VWR, Ince-Line; Schlieren, Switzerland) for 2 days at a temperature of 37°C. When the abrasive particles were dry, they were attached to carbon adhesive tabs (Carbon Adhesive Tabs, Electron Microscopy Science; Hatfield, UK). The tabs were then coated with 10 nm of carbon, and finally SEM (Gemini SEM, Zeiss; Jena, Germany) imaging was performed to determine abrasive particle shape. The magnification used for imaging was 5000X.

### Statistical Analysis

The endpoint ‘abrasive dentin wear’ was plotted against the experimental treatments, i.e. three different types of abrasive particles and two different brushing modes (continuously and intermittently). Likewise, descriptive statistics were calculated (mean and standard deviation). According to the repeated-measures design of the study, a mixed effects linear model was fitted to the data with abrasive dentin wear as the target variable, type of abrasive particles and brushing mode as fixed effects, and a random intercept accounting for the repeated measures of roots. No severe violations of model assumptions could be detected using residual analyses. Marginal means were subsequently estimated and the two brushing modes were statistically compared against each other within each type of abrasive, setting a significance level of α < 0.05 and Kenward-Roger degrees of freedom for the three comparisons.

## Results

### Abrasive Dentin Wear

**Fig 1:**
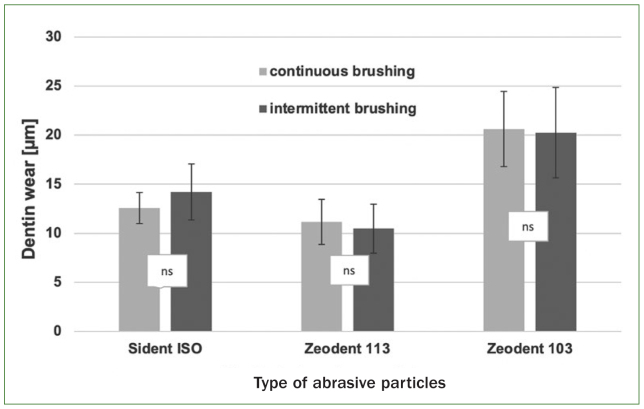
Mean (± SD) abrasive dentin wear for the three types of abrasive particles (Sident 2480-1, Zeodent 113 and Zeodent 103) and the two different brushing modes (continuous and intermittent). Differences within the same type of abrasive particles for the two different brushing protocols that were not statistically significant are marked with ‘ns’.

The abrasive dentin wear for the three types of abrasive particles (Sident 2480-1, Zeodent 113 and Zeodent 103) and the two different brushing modes is presented in Fig 1.

ANOVA showed that neither the brushing mode (p = 0.72) nor the interaction (p = 0.18) of brushing mode and the type of abrasive particles had a statistically significant influence on abrasive dentin wear. Only the type of abrasive particles had a statistically significant influence on abrasive dentin wear (p < 0.001).

Pairwise comparisons of the abrasive dentin wear within the respective types of abrasive particles showed no statistically significant difference for the two different brushing modes (Sident 2480-1: p = 0.089; Zeodent 113: p = 0.480 and Zeodent 103: p = 0.700).

### Scanning Electron Microscopy

**Fig 2:**
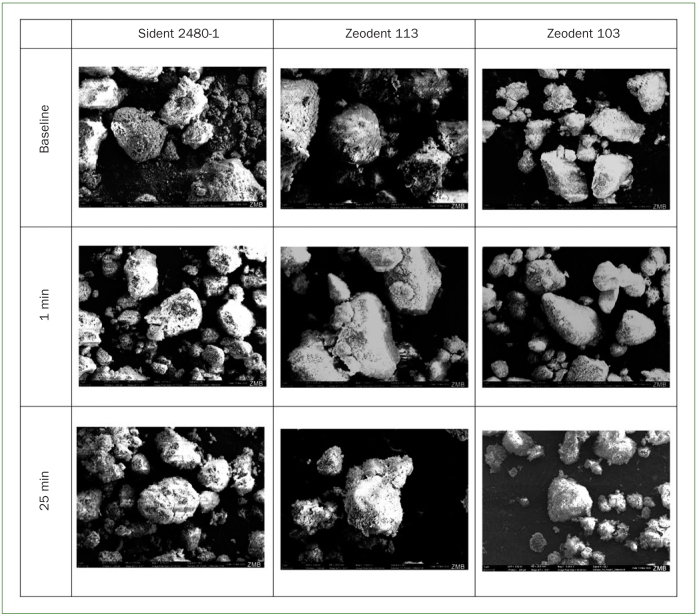
Scanning electron micrographs of the various abrasive particles at baseline and after 1 min and 25 min of brushing (magnification: 5000X).

Scanning electron micrographs of the various abrasive particles at baseline and after 1 min and 25 min of brushing are shown in Fig 2.

SEM images showed a large number of diversities in shape and size within one group of abrasive particles. Therefore, it was not possible to make a statement about the change in shape when the abrasive particles were compared at baseline and after 1 min of brushing or 25 min of brushing. However, it seems that there is no fundamental change in the abrasive particle size and shape during 25 min of brushing.

### pH-Values of Slurries

The pH-values of the slurries measurement at baseline, after 1 min and 25 min of brushing are presented in Table 2.

**Table 2:** pH values of the slurries measured at baseline, after 1 min and 25 min of brushing for the tested abrasives

	Slurries		
	Sident 2480-1	Zeodent 113	Zeodent 103
Baseline	7.17	7.11	7.31
1 min	7.35	7.28	7.30
25 min	7.59	7.44	7.66

At baseline, all slurries showed a nearly neutral pH. Over the 25 min of brushing, a slight increase in pH for all the slurries was observed.

## Discussion

In the present study, bovine teeth were used. But to provide a clinically relevant situation, human teeth are preferable for in vitro and in situ dental research. However, there are some limitations and disadvantages involved in using human teeth. The most common reason for extracting a human tooth is extensive carious lesions or other defects. Therefore, they are often difficult to obtain in sufficient quantity and with adequate quality. Moreover, the morphology of human teeth (relatively small, possessing curved surfaces) also presents some limitations in terms of test requirements. Furthemore, ethical issues could be a reason for finding alternatives to human teeth in dental research.

In this study, permanent bovine incisors were used. Several studies have shown that bovine teeth can be used as a valid substitute for human teeth in dental research.^9,15,16,20^ Bovine teeth are larger, have flatter surfaces, and are easier to obtain than human ones. No statistically significant difference in the susceptibility to toothbrushing abrasion was observed between human and bovine enamel or between human and bovine dentin.^9,15,16^

Previous research shows that storage conditions have influence the profilometrical determination with a stylus profilometer.^2^ Therefore, it was ensured that the specimens used for this study were stored in tap water at all times when not undergoing experiment steps.^2^

In the present study, three different abrasives were tested. They were chosen to cover a wide range of abrasives (RDA 67–174). They were tested in an earlier study performed in our laboratory, and they have been described as chemically inert and biocompatible.^6^

Due to certain inaccuracies and the fact that certain points (e.g. how long can samples be used?) are not addressed in the ISO standards, differences of nearly 100% may occur when the RDA of a toothpaste is measured at 2 different test centers.

In the present study, dentin wear caused by abrasion was recorded using the surface profilometry method with a custom-made software program. Sabrah et al^11^ showed mixed results regarding the application of profilometry for measuring abrasivity of a toothpaste slurry. They observed poor correlations between abrasion measured with a profilometer compared to the RDA method. Specifically, they showed that there is poor correlation between a profilometer and the RDA method using toothpaste, with an RDA value < 50.^11^ However, other studies^1,12,13,17^ have emphasised the usefulness and validity of the profilometry-based abrasivity method and the method described in ISO 10609 Annex B specification.^7^ Thus, use of profilometry in our study followed accepted rationales made for testing abrasivity of toothpaste slurries.

The automatic brushing machine used in our study fulfilled all the requirements described by White et al^17^ and by the ISO 11609 specification.^7^

The pH of the slurries was constantly more or less neutral, although a slight increase over time was recorded when comparing the pH at baseline, after 1 min and 25 min.

In the present study, neither the brushing mode (p = 0.72) nor the interaction (p = 0.18) of the brushing mode and the type of abrasive particles had a statistically significant influence on abrasive dentin wear. Therefore, the null hypothesis of the present study was confirmed. In the SEM analysis, the abrasive particles of the slurries, in which shape is of crucial importance for their abrasivity,^14^ showed no noticeable change in size or shape between baseline, after 1 min and 25 min. This observation might explain why brushing continuously (25 min) or intermittently (25 x 1 min) does not cause a statistically significant difference in abrasive dentin wear. However, it must be taken into consideration that the present study was performed with slurries containing experimental toothpastes. Under clinical conditions, patients use commercially available toothpastes with different abrasives, pHs and a variety of other compounds. Especially when using toothpastes with slightly acidic pHs, brushing continuously might cause different abrasive dentin wear than brushing intermittently, as we found that after 25 min of brushing, the pH of the slurries increased.^18^

In the present study, a brushing force of 2.5 N and a standard toothbrush (PARO 43) was used. However, abrasion is a multifactorial process, and other factors such as erosive and mechanical challenges may enhance dental hard tissue loss. Different brushing forces and materials, shapes and stiffness of toothbrush bristles may also have different impacts on dental hard tissue loss and should therefore also be taken into consideration.^8,19^

## Conclusion

Determination of the abrasiveness of dental toothpaste slurries does not depend on whether these slurries are tested while brushing continuously or intermittently.

## References

[ref1] Addy M (2010). Determination of relative dentifrice abrasivity to enamel and dentine by a surface profile method. J Clin Dent.

[ref2] Attin T, Becker K, Roos M, Attin R, Paqué F (2009). Impact of storage conditions on profilometry of eroded dental hard tissue. Clin Oral Investig.

[ref3] Bächli K, Schmidlin PR, Wegehaupt F, Paqué F, Ramenzoni L, Botter S (2019). Remineralization of artificial dentin caries using dentin and enamel matrix proteins. Materials (Basel).

[ref4] Ghassemi A, Vorwerk L, Hooper W, Patel V, Milleman JL, Milleman KR (2014). Comparative plaque removal efficacy of two new powered toothbrushes and a manual toothbrush. J Clin Dent.

[ref5] González-Cabezas C, Hara AT, Hefferren J, Lippert F (2013). Abrasivity testing of dentifrices – challenges and current state of the art. Monogr Oral Sci.

[ref6] Hamza B, Attin T, Cucuzza C, Gubler A, Wegehaupt FJ (2020). RDA and REA values of commercially available toothpastes utilising diamond powder and traditional abrasives. Oral Health Prev Dent.

[ref7] (2017). ISO ISO 11609: Dentistry – dentifrices – requirements, test methods and marking.

[ref8] Körner P, Inauen DS, Attin T, Wegehaupt FJ (2020). Erosive/abrasive enamel wear while using a combination of anti-erosive toothbrush/-paste. Oral Health Prev Dent.

[ref9] Miller WD (1907). Wasting of tooth tissues variously designated as erosion, abrasion, chemical abrasion, denudation. Dent Cosmos.

[ref10] Philpotts CJ, Weader E, Joiner A (2005). The measurement in vitro of enamel and dentine wear by toothpastes of different abrasivity. Int Dent J.

[ref11] Sabrah AHA, Lippert F, Kelly AB, Hara AT (2013). Comparison between radiotracer and surface profile methods for the determination of dentifrice abrasivity. Wear.

[ref12] Schneiderman E, Colón E, White DJ, St John S (2015). A Profilometry-based dentifrice abrasion method for V8 brushing machines part II: Comparison of RDA-PE and radiotracer RDA measures. J Clin Dent.

[ref13] Schneiderman E, Colón EL, White DJ (2017). A Profilometry-based dentifrice abrasion method for V8 brushing machines part III: Multi-laboratory validation testing of RDA-PE. J Clin Dent.

[ref14] Tawakoli PN, Becker K, Attin T (2018). Abrasive effects of diamond dentifrices on dentine and enamel. Swiss Dent J.

[ref15] Wegehaupt FJ, Gries D, Wiegand A, Attin T (2008). Is bovine dentine an appropriate substitute for human dentine in erosion/abrasion tests?. J Oral Rehabil.

[ref16] Wegehaupt FJ, Widmer R, Attin T (2010). Is bovine dentine an appropriate substitute in abrasion studies?. Clin Oral Investig.

[ref17] White DJ, Schneiderman E, Colón E, St John S (2015). A profilometry-based dentifrice abrasion Method for V8 brushing machines. Part I: Introduction to RDA-PE. J Clin Dent.

[ref18] Wiegand A, Attin T (2011). Design of erosion/abrasion studies – insights and rational concepts. Caries Res.

[ref19] Wiegand A, Kuhn M, Sener B, Roos M, Attin T (2009). Abrasion of eroded dentin caused by toothpaste slurries of different abrasivity and toothbrushes of different filament diameter. J Dent.

[ref20] Yassen GH, Platt JA, Hara AT (2011). Bovine teeth as substitute for human teeth in dental research: a review of literature. J Oral Sci.

